# New Submicron Low Gate Leakage In_0.52_Al_0.48_As-In_0.7_Ga_0.3_As pHEMT for Low-Noise Applications

**DOI:** 10.3390/mi12121497

**Published:** 2021-11-30

**Authors:** Mohamed Fauzi Packeer Mohamed, Mohamad Faiz Mohamed Omar, Muhammad Firdaus Akbar Jalaludin Khan, Nor Azlin Ghazali, Mohd Hendra Hairi, Shaili Falina, Mohd Syamsul Nasyriq Samsol Baharin

**Affiliations:** 1 School of Electrical and Electronic Engineering, Universiti Sains Malaysia, Nibong Tebal 14300, Pulau Pinang, Malaysia; firdaus.akbar@usm.my (M.F.A.J.K.); azlin.ghazali@usm.my (N.A.G.); 2 Collaborative Microelectronic Design Excellence Center (CEDEC), Universiti Sains Malaysia, Sains@USM, Bayan Lepas 11900, Pulau Pinang, Malaysia; faiz_omar@usm.my (M.F.M.O.); shailifalina@moegi.waseda.jp (S.F.); 3 Faculty of Electrical Engineering, Technical University of Malaysia Malacca, Durian Tunggal 76100, Melaka, Malaysia; hendra@utem.edu.my; 4 Faculty of Science and Engineering, Waseda University, Tokyo 169-8555, Japan; 5 Institute of Nano Optoelectronics Research and Technology (INOR), Universiti Sains Malaysia, Sains@USM, Bayan Lepas 11900, Pulau Pinang, Malaysia

**Keywords:** 2DEG, III-V material, InAlAs, InGaAs, InP, LNA, low temperature (LT), MBE, MMIC, pHEMT, semiconductor device

## Abstract

Conventional pseudomorphic high electron mobility transistor (pHEMTs) with lattice-matched InGaAs/InAlAs/InP structures exhibit high mobility and saturation velocity and are hence attractive for the fabrication of three-terminal low-noise and high-frequency devices, which operate at room temperature. The major drawbacks of conventional pHEMT devices are the very low breakdown voltage (<2 V) and the very high gate leakage current (∼1 mA/mm), which degrade device and performance especially in monolithic microwave integrated circuits low-noise amplifiers (MMIC LNAs). These drawbacks are caused by the impact ionization in the low band gap, i.e., the In${}_{x}$Ga${}_{\left(1-x\right)}$As (*x* = 0.53 or 0.7) channel material plus the contribution of other parts of the epitaxial structure. The capability to achieve higher frequency operation is also hindered in conventional InGaAs/InAlAs/InP pHEMTs, due to the standard 1 μm flat gate length technology used. A key challenge in solving these issues is the optimization of the InGaAs/InAlAs epilayer structure through band gap engineering. A related challenge is the fabrication of submicron gate length devices using I-line optical lithography, which is more cost-effective, compared to the use of e-Beam lithography. The main goal for this research involves a radical departure from the conventional InGaAs/InAlAs/InP pHEMT structures by designing new and advanced epilayer structures, which significantly improves the performance of conventional low-noise pHEMT devices and at the same time preserves the radio frequency (RF) characteristics. The optimization of the submicron T-gate length process is performed by introducing a new technique to further scale down the bottom gate opening. The outstanding achievements of the new design approach are 90% less gate current leakage and 70% improvement in breakdown voltage, compared with the conventional design. Furthermore, the submicron T-gate length process also shows an increase of about 58% and 33% in f${}_{T}$ and f${}_{max}$, respectively, compared to the conventional 1 μm gate length process. Consequently, the remarkable performance of this new design structure, together with a submicron gate length facilitatesthe implementation of excellent low-noise applications.

## 1. Introduction

In the field of RF and microwave design and for any transistor technology in the frequency range 2–1000 GHz, the lowest possible noise figures are achieved by the InP-based high electron mobility transistor (HEMT) [1], which has proven itself to be the best performing three-terminal device [2,3]. However, amongst all group III-V material systems, the performance of the InP-based HEMT is enhanced by the use of strained high mobility InGaAs material, where the lowest noise figures and highest-frequency performance can be realized. The conventional lattice matched In${}_{0.53}$Ga${}_{0.47}$As–In${}_{0.52}$Al${}_{0.48}$As pseudomorphic HEMT (pHEMT) on an InP substrate, incorporating an In${}_{0.7}$Ga${}_{0.3}$As compressively strained channel, is an excellent choice for fabricating high-frequency low-noise devices, owing in part to its high mobility and high saturation velocity due to the high indium content in the InGaAs channel [4,5]. Unfortunately, a conventional low-noise pHEMT employing this material system experiences a low breakdown voltage of about 2–4 V and a high gate leakage of around 1 mA mm${}^{-1}$ at −5 V [6,7].

Over the years as reported, a lot of effort was put in to improve the basic pHEMT design such as the use of platinum gate metal [8] as the Schottky barrier, instead of the commonly used titanium, a much higher InGaP and AlAs spacer layer [9,10], a composite channel design [11] and double-recessed structures [12,13]. Unfortunately, most of these approaches degrade considerably the unity current gain cut-off frequency f${}_{T}$ and increase the noise. Hence, the focus of this study is on the area of epitaxial design, fabrication, characterization and final device performance of new optical sub-μm gate length InP-based pHEMT using InGaAs-InAlAs material systems. The modification of the epitaxial layer design by incorporating a low temperature (LT) In${}_{0.52}$Al${}_{0.48}$As buffer (grown at <200 °C) significantly improves upon the conventional low-noise pHEMT [14], which suffers from high gate current leakage and low breakdown voltage. In addition, a new submicron T-gate process technique using conventional I-line optical lithography is capable of shrinking the gate length from 1 to 0.5 μm by solvent reflow at low temperature (<50 °C) with a high throughput compared to e-beam lithography.

Upon successful completion of the fabrication and characterization of such a structure, there was an observed breakdown voltage and gate leakage improvements of over 70% and 90%, respectively, compared to the conventional InGaAs-InAlAs pHEMT device. These are large improvements in making these devices suitable for low-noise applications. Furthermore, the cut-off frequency is much higher, i.e., 50 GHz compared to only 21.6 GHz of a 1 μm gate length and 200 μm gate width device. Hence, this structure together with submicron gate length could demonstrate high breakdown coupled with ultrahigh speed and a low-noise device.

## 2. Samples: Device Material Epitaxial Growth

Two structures of epilayer were studied and compared, which were grown from an InGaAs/InAlAs/InP family. These two epitaxial layer structures were fully grown using in-house molecular beam epitaxy (MBE) on a RIBER V100 System and carry the prefix XMBE. One of the epitaxial structures, known as XMBE171, was set as the baseline for this study and is identical to the conventional pHEMT. The other epitaxial structure is identified as XMBE56 and is known as the improved pHEMT.

**Figure 1:**
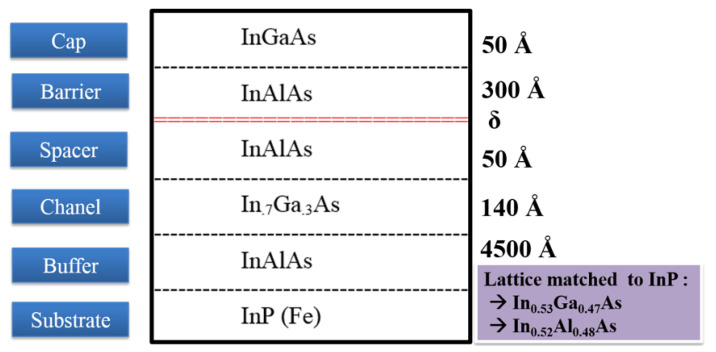
XMBE171 (conventional pHEMT) epitaxial layer grown on in-house RIBER V100 MBE (thickness not to scale).

**Figure 2:**
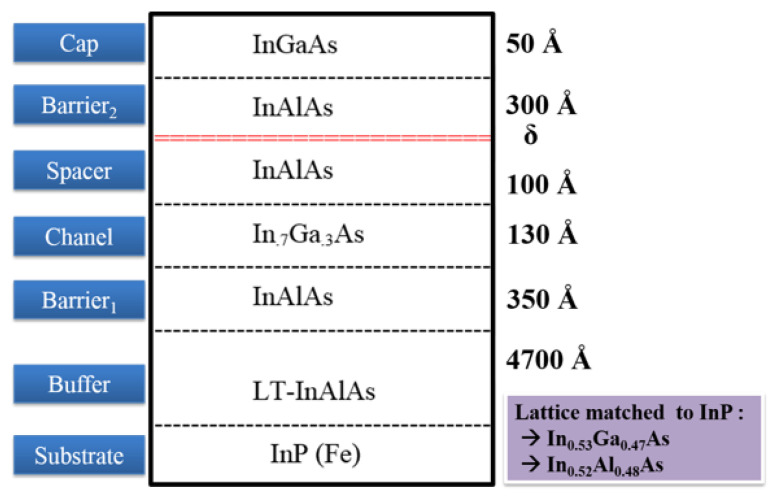
XMBE56 (improved pHEMT incorporating LT-In${}_{0.52}$Al${}_{0.48}$As buffer) epitaxial layer grown on in-house RIBER V100 MBE (thickness not to scale).

Both of XMBE171 and XMBE56 epitaxial layers are grown on a semi-insulating iron-doped InP substrate, i.e., InP (Fe); however, all the epitaxial layers are not doped except for the delta-doped layer. Moreover, the delta-doped is silicon-doped modulation layer with XMBE171 doping is 5.0 × 10${}^{12}$ cm${}^{-2}$ and XMBE56 is 3.6 × 10${}^{12}$ cm${}^{-2}$. As for the growth temperature, the standard growth temperature for all XMBE171 and XMBE56 epitaxial layers are at ∼450 °C, except for LT-In${}_{0.52}$Al${}_{0.48}$As XBE56 being grown at <200 °C. This LT layer increases the resistivity and enhances the surface morphology of the epitaxial/substrate interface, which later improves the off-state and on-state gate current leakages. Detailed overviews of both lattice matched InGaAs/InAlAs/InP pHEMTs are shown in Figure 1 and Figure 2, while Table 1 shows the calculations [15] of the approximate band energies (Eg) and band gap differences (ΔEg) of XMBE171 and XMBE56.

As can be seen in Table 1, the conduction band gap differences or discontinuities are the same for both devices, since both have a lattice-matched In${}_{0.52}$Al${}_{0.48}$As spacer layer to an In${}_{0.7}$Ga${}_{0.3}$As channel.

### Hall Effect Measurements and Band Diagrams

The epilayer structures for both XMBE171 and XMBE56 were characterized through Hall effect measurements, and the results for both are recorded in Table 2. Furthermore, the 2DEG (two-dimensional electron gas) performance—i.e., the carrier mobility and sheet carrier concentration—is compared both at room temperature 300 K and at 77 K. Further data in Table 2 again demonstrate that the sheet carrier concentration of XMBE56 is lower than the XMBE171. This same trend is due to the spacer layer thickness of the XMBE56 device being half of that of the XMBE171 epilayer structure, which can clearly be seen by referring to Figure 1. On the other hand, the carrier mobility for the XMBE56 is higher than the XMBE171 epilayer structure. Hence, the thinner spacer enhances more electrons to be trapped in the 2DEG channel, giving higher carrier concentration, at the same time achieving a stronger Coulomb scattering effect between the δ-doped donor at the barrier layer with the channel, which causes degradation in carrier mobility in the 2DEG channel [16].

Significantly, the excellent carrier transport properties shown for the XMBE56 at room temperature, i.e., with channel carrier concentration = 2.47 × 10${}^{12}$ cm${}^{-2}$ and mobility = 13,169 (cm${}^{2}$/Vs), are important for realizing high speed device fabrication.

**Figure 3:**
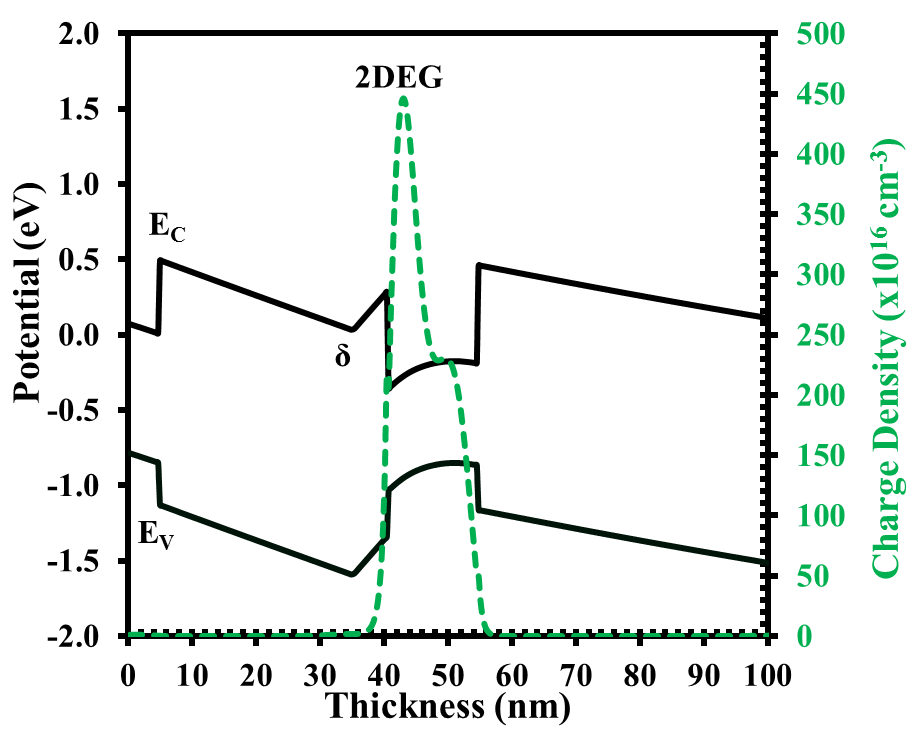
XMBE171 conduction band diagram and electron distribution.

**Figure 4:**
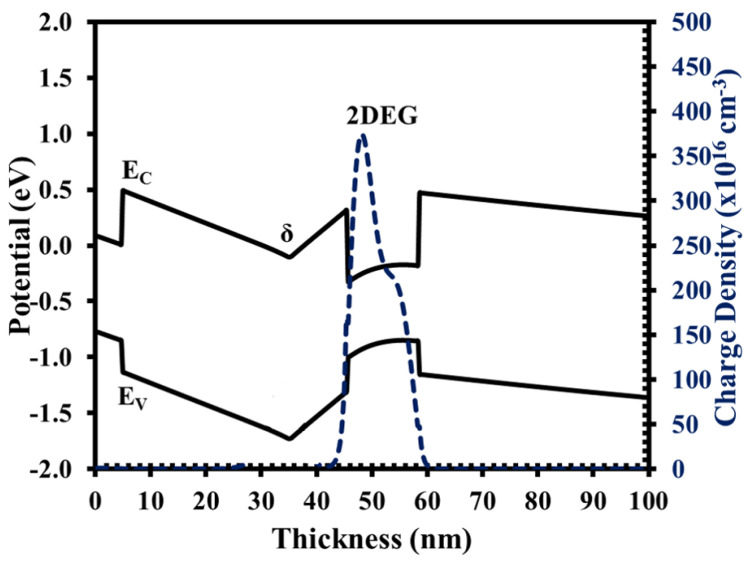
XMBE56 conduction band diagram and electron distribution.

To complete the comparisons, the benefits of using the XMBE56 epitaxial layer can be seen in the WinGreen^®^ band diagram simulation. The illustrations of the energy band diagram for both structures are shown in Figure 3 and Figure 4.

## 3. Device Fabrication

The fabrication process flow and steps of the device using XMBE171 (conventional epitaxial structure) was incorporated with a standard 1 μm process flow. Generally, the flow starts with MESA isolation followed by the selective succinic acid etching of the MESA’s side walls. Then, the thermal evaporation of 50 nm AuGe and 100 nm Au is performed to form source and drain connections on the In${}_{0.53}$Ga${}_{0.47}$As cap layer. To achieve excellent Ohmic contact, the device is then alloyed at 280 °C for 90 s in the N${}_{2}$ environment furnace to allow adequate metal diffusion deep into the 2DEG. The following steps are succinic acid gate recess etching and then definition of the Schottky gate contact on the In${}_{0.52}$Al${}_{0.48}$As barrier layer using a flat gate length process. Finally, the thermal evaporation of 50 nm of Ti and 450 nm Au bond pad metals is performed.

The improved epitaxial structure in this study, XMBE56, is made for a submicron T-gate device. The process starts with the deposition of 200 nm PECVD Si${}_{3}$N${}_{4}$ as a hard mask on the cap layer and then a 1 μm gate opening as for XMBE171, optically defined by I-line lithography and patterned on the hard layer. Next, a soft reflow technique is employed, to reduce the 1 μm gate opening to roughly 0.4 μm. The soft reflowed photoresist is then etched with CF${}_{4}$ plasma for pattern transfer, defining the submicron bottom gate footprint. Upon successful definition of the bottom gate footprint, the MESA and Ohmic steps are similar to the fabrication of XMBE171.

**Figure 5:**
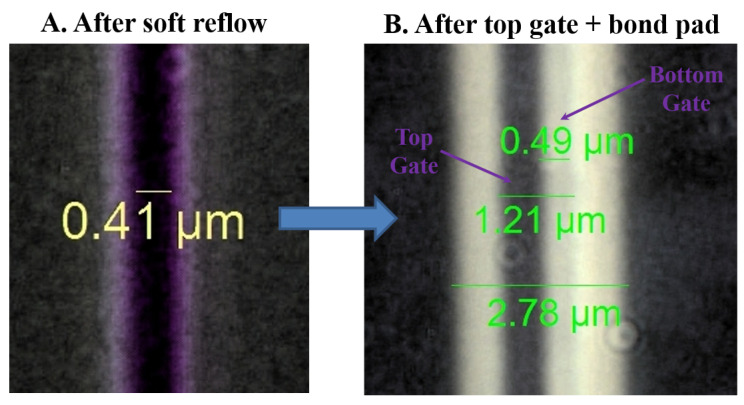
T-gate device fabrication by improved soft reflow chamber setup.

The last step for this submicron fabrication is to metalize the top gate and bond pads; this is different from XMBE171. In this process, the top gate and bond pads are fabricated together in one mask step. Here, the 1 μm gate plus the bond pads opening are again produced through an optical I-line process. Prior to metallization, the gate recess is produced in a similar way to XMBE171. The completed submicron T-gate device is then ready for DC and RF characterization after thermal evaporation of 50 nm Ti then 450 nm Au. Figure 5 shows the XMBE56 device after soft reflow and the T-gate structure.

## 4. Results and Discussion

### 4.1. Direct Current (DC) Characteristics

The TLM measurement was taken at room temperature directly after the Ohmic step and alloying. The data for both devices are recorded in Table 3.

In observing the data in the above table, one could appreciate that the R${}_{sh}$ of the XMBE56 is lower by 14.8% compared to the XMBE171, due to the higher mobility of electrons, by referring to the room temperature Hall effect measurements data in Table 2, i.e., 13,169 and 10,653 cm${}^{2}$/V·s for XMBE56 and XMBE171, respectively. Both devices have a low R${}_{c}$, i.e., <0.2 Ω/mm, demonstrating good Ohmic contact and reflecting deep diffusion of AuGe/Au metals into the 2DEG channel.

Hereafter, the DC characteristics for the devices studied are measured with a cascade ground-signal-ground (G-S-G) 3 pin probe station. The HP-Agilent 4142B Modular DC parameter analyzer and an Agilent Vector Network Analyzer (VNA) connected to the probes were used to measure the devices via their bond pads for data extraction. Furthermore, these two types of pHEMT devices are measured at room temperature, with data averaged over two devices of each type, while the values are normalized to the device gate width for fair comparison. Moreover, data for both the XMBE56 and XMBE171 are compared on only two gate fingers of 2 × 100 μm (200 μm) gate width with 5 μm source-drain separation.

**Figure 6:**
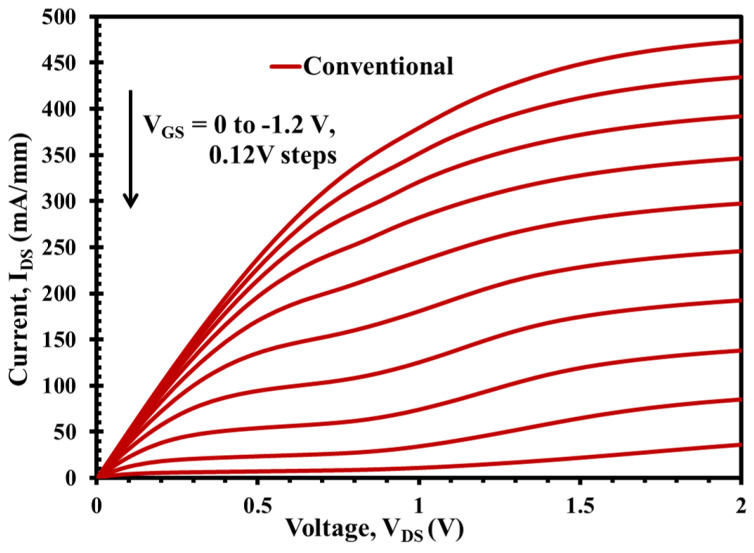
XMBE171 (conventional pHEMT) DC output characteristic with 1 μm length multigate of 2 × 100 μm width. I${}_{DS}$ vs. V${}_{DS}$ when V${}_{GS}$ swept from 0 to −1.2 V.

**Figure 7:**
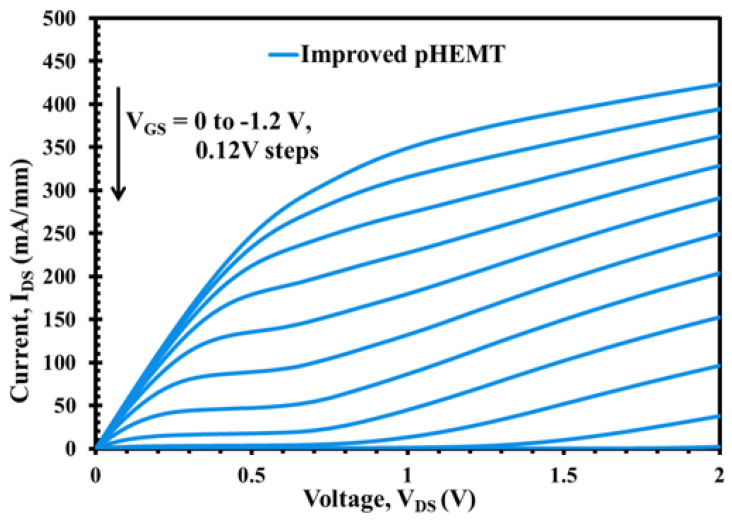
XMBE56 (improved pHEMT) DC output characteristic with 0.5 μm length multigate of 2 × 100 μm width. I${}_{DS}$ vs. V${}_{DS}$ when V${}_{GS}$ swept from 0 to −1.2 V.

The DC output characteristics or I-V curves at various V${}_{GS}$ points from V${}_{GS}$ = 0 V to V${}_{GS}$ = −1.2 V, for both XMBE171 and XMBE56 epilayer structures, are shown in Figure 6 and Figure 7. Both figures show well-behaved curves with sharply defined pinch-off characteristics at room temperature, demonstrating excellent device operation. Analysis from both the curves show that the normalized I${}_{DS}$ current of the XMBE56 is lower than the XMBE171 at the same V${}_{GS}$ biasing, resulting from the lower carrier concentration in the 2DEG channel in the XMBE56 epilayer structure and hence less electron contribution to current conduction, and thus decreased the I${}_{DS}$ output current. Correspondingly, the maximum drain current density (I${}_{DSS}$) at V${}_{GS}$ = 0 V and V${}_{DS}$ = 1 V is 350 and 370 mA/mm for XMBE56 and XMBE171, respectively.

More insight into XMBE56 I-V curve shows a significant kink effect and high output conductance compared to the XMBE171. The origin of the kink effect was first linked with traps in the InAlAs buffer layer, where the traps capture energetic electrons and release them when drain bias increases. Hence, the initial efforts to eliminate kink focused on improving the quality of InAlAs buffer layer, which in this case incorporates LT buffer in XMBE56 to lower the trap density and to control the electron trapping behavior. Thus, the LT buffer should be able to eliminate the kink effect but, in the case of XMBE56, is due to the effect of the shorter gate length, which also tends to allow more electrons to be injected into the buffer layer [17].

**Figure 8:**
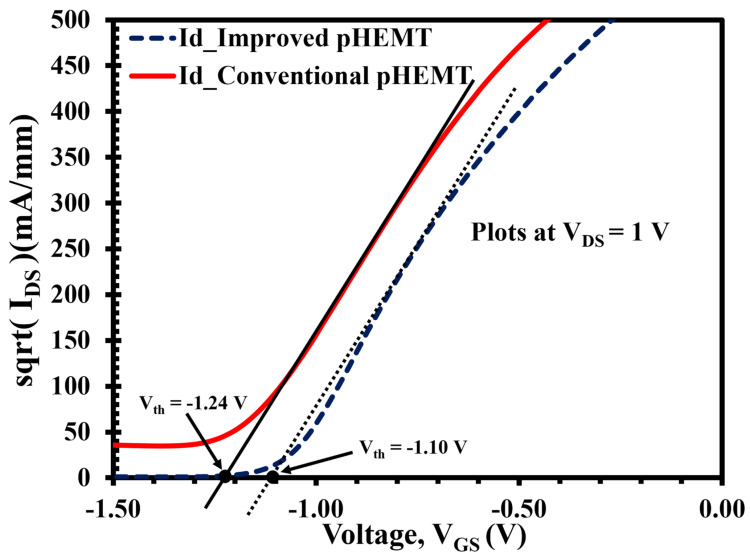
XMBE171 (conventional pHEMT) and XMBE56 (improved pHEMT) threshold voltage (V${}_{th}$) at V${}_{DS}$ = 1 V with 2 × 100 μm gate width.

The threshold voltages (V${}_{th}$) for both devices are shown in Figure 8. This is an important parameter for the operation of a pHEMT and is determined by the thickness of the higher bandgap barrier layer [18], i.e., In${}_{0.52}$Al${}_{0.48}$As, or more specifically, the gate to channel distance when the In${}_{0.53}$Ga${}_{0.47}$As cap layer is removed after gate recessing. Given these points, the V${}_{th}$ for the LT-In${}_{0.52}$Al${}_{0.48}$As buffer of XMBE56 and XMBE171 are approximately −1.10 and −1.24 V, respectively. These are not significantly different, because the difference in gate-to-channel distance between the devices is only 50 Å difference, referring to the epitaxial structures shown in Figure 1 and Figure 2.

**Figure 9:**
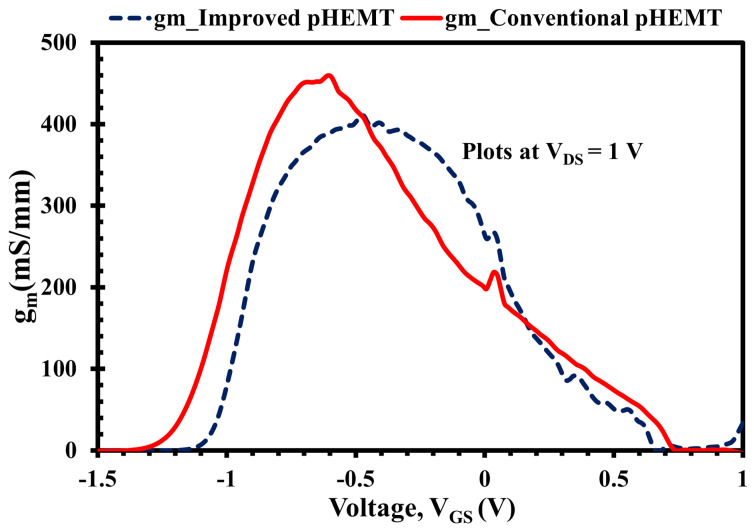
XMBE171 (conventional pHEMT) and XMBE56 (improved pHEMT) extrinsic transconductance (g${}_{m}$) at V${}_{DS}$ = 1 V with 2 × 100 μm gate width.

In addition, Figure 9 shows the change of extrinsic transconductance (g${}_{m}$) as a function of V${}_{GS}$ for both pHEMT devices, under the biasing of V${}_{DS}$ = 1 V. The peak from each plot is the maximum g${}_{m}$ value where XMBE56 peak is at V${}_{GS}$ = −0.46 V with g${}_{mmax}$ = 403 mS/mm and XMBE171 peak is at V${}_{GS}$ = −0.58 V with g${}_{mmax}$ = 450 mS/mm.

Moreover, there are two essential observations from Figure 9. The first observation is that the XMBE56 g${}_{mmax}$ is 11.7% lower compared to the XMBE171, even though the XMBE56 has a 50% shorter gate (0.5 μm vs. 1 μm). This is due to the fact that the extrinsic transconductance (g${}_{m}$) of pHEMT devices is inversely proportional to the gate to channel distance [19], where the XMBE171 distance is 50 Å shorter. Referring to Figure 1 and Figure 2, the difference in the gate-to-channel distance is given by the spacer layer thickness, whereby the spacer layer for XMBE56 is thicker than the spacer layer of the XMBE171, hence reducing the electron transfer efficiency from the δ-doped donor layer into the 2DEG quantum well. The impact of the thicker spacer layer of the XMBE56 can be seen from the Hall effect measurements data in Table 2, where the 2DEG sheet carrier concentration of the XMBE56 is 22% lower compared to the conventional XMBE171. Note that it is important to maximize the 2DEG sheet carrier concentration to achieve large transconductance as well as large drive currents [18]. Furthermore, the transconductance is also related directly to the saturation velocity (V${}_{sat}$) and gate capacitance (C${}_{g}$). As the channel material of the XMBE56 and XMBE171 are similar, i.e., In${}_{0.7}$Ga${}_{0.3}$As, their V${}_{sat}$ values are also similar, i.e., 2.6 ± 0.2 × 10${}^{7}$ cm/s [20,21] while the C${}_{g}$ for the XMBE171 should be higher compared to the XMBE56. The fact that C${}_{g}$ for the XMBE171 is higher than the XMBE56 is due to the shorter gate-to-channel distance and the larger gate length L${}_{g}$. In short, the extrinsic transconductance for the XMBE56 is lower than that for the XMBE171, mainly due to the impact of gate to channel distance and C${}_{g}$, while the shorter gate length and V${}_{sat}$ do not seem to have much effect. On the other hand, the lower saturation current (I${}_{DSS}$) and lower carrier concentration sheet carrier density mentioned leads to lower g${}_{m}$ in the XMBE56.

The second observation is that the shift in the two plots is consistent with the shift in the V${}_{th}$ in Figure 8, for the same reason—i.e., the influence of gate-to-channel distance.

**Figure 10:**
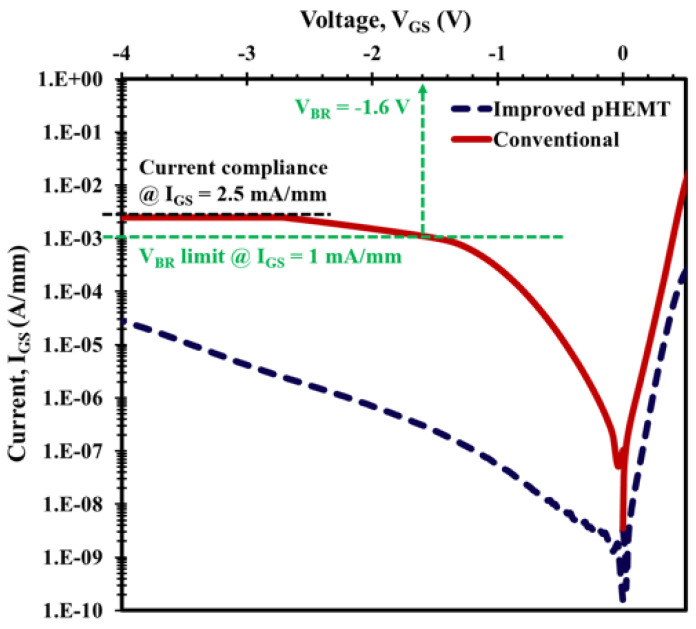
XMBE171 (conventional pHEMT) and XMBE56 (improved pHEMT) off-state Schottky gate leakage current at V${}_{GS}$ = −4 V.

**Figure 11:**
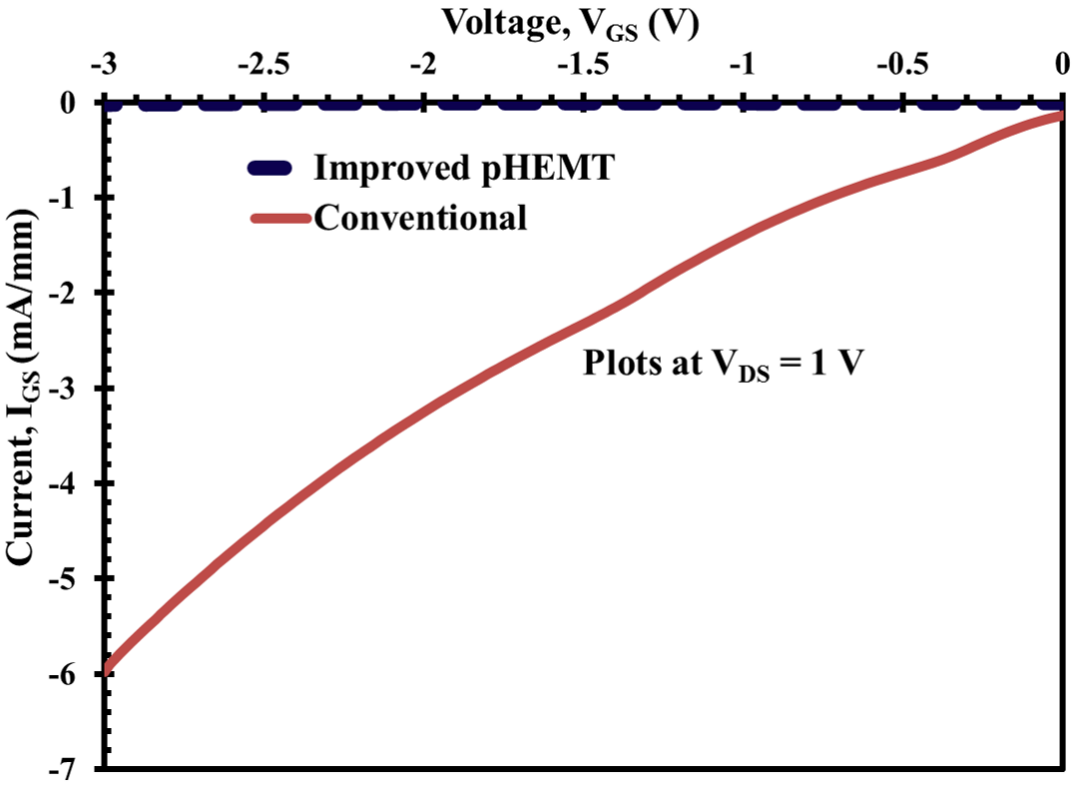
XMBE171 (conventional pHEMT) and XMBE56 (improved pHEMT) on-state Schottky gate leakage current at V${}_{GS}$ = −3 V, in mA-range.

In the DC characteristics presented in Figure 10 and Figure 11, significant advantages can be seen in the XMBE56 over XMBE171. In Figure 10, the off-state Schottky gate leakage current, the reverse bias curves for the XMBE56 gate current leakage, is −28 μA/mm @ V${}_{GS}$ = −4 V, compared to −2500 μA/mm or −2.5 mA/mm at the same V${}_{GS}$ biasing for the XMBE171. This is better than a 99% reduction, which could result in an excellent low-noise device. In addition, at a reference gate current I${}_{GS}$ of 1mA/mm, the XMBE56 off-state breakdown voltage (V${}_{BR}$) is higher than that of XMBE171. The extrapolation of XMBE56 at the reference I${}_{GS}$ gives roughly a V${}_{BR}$ of −6 V compared to −1.6 V for XMBE171; this is about a 73% improvement in V${}_{BR}$ compared to the XMBE171 and also other reported research work [22,23,24]. The benefit of this high-breakdown voltage of XMBE56 could also make it a good low-noise amplifier in a receiver, while requiring minimal protection circuits.

The forward bias curves in Figure 10 are important in extracting the Schottky diode characteristics of the devices—the barrier height ($\Phi_{B}$) and ideality factor (η). XMBE56 has a $\Phi_{B}$ of 0.53 eV and η of 1.37, thus demonstrating a good Schottky contact. Likewise, the extracted values for XMBE171 have a $\Phi_{B}$ of 0.50 eV and η of 1.60, which are substantially degraded compared to the XMBE56.

**Figure 12:**
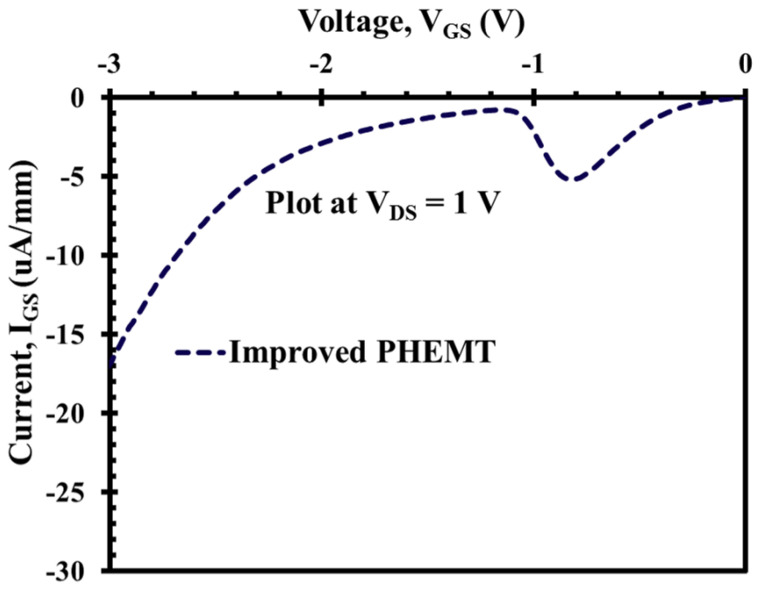
XMBE56 (improved pHEMT) on-state Schottky gate leakage current, in μA-range.

Another key advantage of the XMBE56, as previously mentioned, is shown in Figure 11 and Figure 12; these figures show exceptional results for the on-state gate leakage current when compared to XMBE171. At biasing V${}_{DS}$ = 1 V, the on-state Schottky gate current leakage, which is caused by impact ionization, is only about −5.2 μA/mm compared to −2000 μA/mm (or −2 mA/mm) for XMBE171. Under the same circumstances, at V${}_{GS}$ = −3 V, XMBE56 also shows very low reverse Schottky gate current leakage due to electron tunneling, i.e., −17 μA/mm, whereas XMBE171 has a very high value of −6000 μA /mm (or −6 mA/mm).

As noted from the performance of both the off-state and on-state Schottky gate current leakage of XMBE56, the excellent results are due to modification of the epitaxial layer design, not only the low temperature (LT) In${}_{0.52}$Al${}_{0.48}$As buffer (grown at <200 °C), but also the thicker spacer, which gives a remarkable improvement over the conventional low-noise pHEMT.

The thicker spacer used in XMBE56 reduces the efficiency of electron transfer from the donor/supply layer to the 2DEG. For this reason, the concentration of electrons in the 2DEG is less, consequently lowering the rate of impact ionization in the channel. Furthermore, the thicker spacer is also capable of reducing the number of holes—generated from the impact ionization process in the channel—from being collected by the gate, which is negatively biased. Moreover, the mechanism to reduce both the off-state and on-state gate current leakages in XMBE56 is through the improvement of the buffer layer. The leakage mechanism is also contributed by the buffer layer; hence, one of the methods for buffer layer optimization is by using low temperature (LT) buffers. The LT buffer gives high resistivity [25,26,27,28] and reduces the injection of electrons from the channel into the buffer and substrate, and undesirable electron flow from source to drain in the buffer and substrate. As these electrons in the buffer and substrate are far from the gate, where the gate modulation is inefficient, the reduction of these leakages through the buffer and substrate lowers the excess in drain current and suppresses the reverse gate leakage current, and it also increases the V${}_{BR}$ [27,29].

The holes that are generated from impact ionization can create a leakage path inside the buffer; hence, these holes have a positive fixed charge which enhances the injection of hot electrons to the channel and buffer and increases the reverse gate leakage current [27,30]. The high resistivity of the LT buffer also helps to make the electrons from source to drain more confined in the channel layer [31] and reduces leakage to the buffer. Furthermore, the In${}_{0.52}$Al${}_{0.48}$As Barrier${}_{1}$ layer, grown after the LT-In${}_{0.52}$Al${}_{0.48}$As buffer (refer Figure 2 on the XMBE56 epitaxial layer), does not use the super lattice (SL) structure, as previously performed by other researchers [28,32]. Without using SL, which is well known to smoothen the layer surface and stop defect threading, XMBE56 epilayer still exhibits attractive pHEMT device features and can be used in low-noise millimeter-wave MMICs (monolithic microwave integrated circuits) and high-power millimeter-wave application devices.

### 4.2. Radio Frequency (RF) Characteristics

The extraction of the small signal S-parameters through RF measurement at room temperature was performed for both the XMBE56 and XMBE171 pHEMTs. The DC measurement was performed using a cascade ground-signal-ground (G-S-G) 3-pin probe station which was connected to an HP-Agilent 4142B Modular DC Source. The metallization for the bond pads was 50 nm Ti followed by 450 nm Au—the same as for the gate. The pHEMT devices were measured at room temperature on two-gate-fingered devices, with 2 × 100 μm (200 μm) gate width and 5 μm source-drain separation. The RF characteristics of both of the measured devices were extracted under biasing at each device’s maximum or peak g${}_{m}$. Details of the RF biasing values are summarized in Table 4.

**Figure 13:**
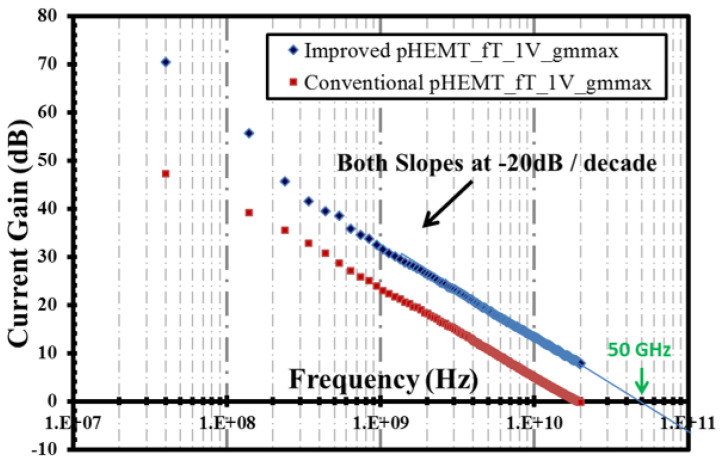
Unity current gain or cut-off frequency (f${}_{T}$) at g${}_{mmax}$ for V${}_{DS}$ = 1 V biasing.

**Figure 14:**
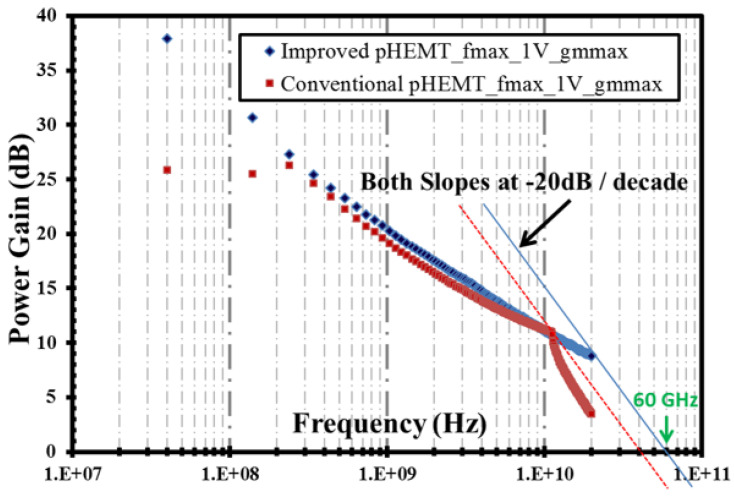
Unilateral power gain or maximum frequency (f${}_{max}$) at g${}_{mmax}$ for V${}_{DS}$ = 1 V biasing.

The results from the RF measurement are shown in Figure 13 and Figure 14 for the cut-off frequency (f${}_{T}$) and the maximum frequency (f${}_{max}$), respectively. Note that the VNA used in this RF measurement has the capability to sweep frequency only until 20 GHz; hence, for the submicron device, i.e., the XMBE56, the values of f${}_{T}$ and f${}_{max}$ were extrapolated with a slope of −20 dB/decade.

A high f${}_{T}$ of 50 GHz was observed for the XMBE56 device, compared with the XMBE171. The improvement is about a 58% increase in f${}_{T}$, due to the employment of a 0.5 μm submicron T-gate length in XMBE56, compared to the 1 μm flat gate length in XMBE171. It is important to note that, even with lower output current (I${}_{DS}$) and transconductance (g${}_{m}$) as shown in the DC characteristics, the XMBE56 device can still achieve a very high f${}_{T}$ compared to XMBE171. The improvement is significant for XMBE56, due to the incorporation of a “new optically-defined submicron gate length” method, which is able to shrink the gate length by roughly 50%. Equation (1) proves that the f${}_{T}$ is strongly dependent on the gate length, i.e., the shorter the gate length, the higher the f${}_{T}$.

(1) $$f_{T}=\frac{v_{sat}}{2\pi L_{g}}$$

where $v_{sat}$ is the electron saturation velocity and $L_{g}$ is the gate length.

In the same manner, the maximum frequency (f${}_{max}$) of the XMBE56 submicron T-gate length device is also improved. The f${}_{max}$ is 60 GHz, which is about a 33% increase compared to the 1 μm flat gate length in XMBE171. It is important to realize that, unlike f${}_{T}$, the parasitic elements such as R${}_{G}$ (gate resistance) and C${}_{GD}$ (gate-drain capacitance) play an important role in f${}_{max}$ [33,34]. Hence, the incorporation of T-shaped gate for the submicron gate length helps to minimize the parasitic elements, which is important for the future fabrication of low-noise amplifiers as it allows the noise figure to be minimized.

## 5. Conclusions

The practicality of this optimized soft reflow process was realized through the fabrication of a T-gate pHEMT device, which for this study has a bottom gate footprint of 0.5 μm. The next part focuses on the design of an epitaxial structure, fabrication and characterization of a 0.5 μm T-gate length InP-based pHEMT incorporating InGaAs-InAlAs material systems. The purpose of this part of study is to improve the conventional 1 μm flat gate length InGaAs/InAlAs/InP pHEMT, reducing the high gate leakage current and improving the low breakdown voltage, which impede its performance in low-noise devices.

This study confirms that, by employing a low temperature (LT) In${}_{0.52}$Al${}_{0.48}$As buffer (grown at <200 °C), together with the thicker spacer in the new design, the improved XMBE56 demonstrated a significant enhancement in the breakdown voltage and reduction in off-state and on-state Schottky gate leakage current. The DC characteristics, upon successful completion of the fabrication and characterization of improved structure, demonstrate breakdown voltage and gate current leakage improvements of over 70% and 90%, respectively, compared to the XMBE171 InGaAs-InAlAs pHEMT device. These are significant improvements that make this device suitable for low-noise applications.

The new optically defined submicron gate length process using conventional I-line optical lithography also indicates an increase of about 58% and 33% in f${}_{T}$ and f${}_{max}$, respectively, compared to the XMBE171 with a 1 μm gate length and 200 μm gate width. Consequently, this improved structure, together with a submicron gate length, demonstrated a device breakthrough with a high breakdown voltage coupled with ultrahigh speed and low noise.

## Figures and Tables

**Table 1:** XMBE171 and XMBE56 band energies (Eg) and band gap difference (ΔEg).

Device	Wide Band Gap1	Narrow Band Gap2	Eg1 (eV)	Eg2 (eV)	ΔEg (eV)
XMBE171	In${}_{0.52}$Al${}_{0.48}$As	In${}_{0.7}$Ga${}_{0.3}$As	1.4	0.579	0.821
XMBE56	In${}_{0.52}$Al${}_{0.48}$As	In${}_{0.7}$Ga${}_{0.3}$As	1.4	0.579	0.821

Eg = band energies, ΔEg = band gap difference, eV = electron volt, InAlAs = indium aluminium arsenide, InGaAs = indium gallium arsenide.

**Table 2:** XMBE171 and XMBE56 epilayer hall effect measurements data.

Measurement	XMBE171	XMBE56
Sheet Carrier Concentration (n${}_{H}$) at 300 K/77 K ( × 10${}^{12}$ cm${}^{-2}$)	3.16/3.56	2.47/2.61
Hall Mobility ($\mu_{H}$) at 300 K/77 K (cm${}^{2}$/V·s)	10,653/24,649	13,169/42,906

K = kelvin, cm = centimetre, V = volt, s = second.

**Table 3:** Devices comparison on R${}_{sh}$ and R${}_{c}$ from TLM measurement.

Parameter	XMBE171	XMBE56
R${}_{sh}$ (Ω/mm)	182	155
R${}_{c}$ (Ω.mm)	0.19	0.13

R${}_{sh}$ = sheet resistance, R${}_{c}$ = contact resistance, Ω = ohm, mm = millimetre, TLM = transfer length method.

**Table 4:** RF biasing conditions for XMBE56 (improved) and XMBE171 (conventional).

Parameter	XMBE56	XMBE171
g${}_{mmax}$ (mS/mm)	410 ± 5	459 ± 5
V${}_{DS}$ (V)	1.0	1.0
V${}_{GS}$ (V)	−0.47 ± 5	−0.61 ± 5
I${}_{DS}$ (mA/mm)	171.5 ± 1	173.3 ± 1

mS = millisiemens, V = volt, mA = milliampere, mm = millimeter; g${}_{m}$ = transconductance, V${}_{DS}$ = drain-source voltage, V${}_{GS}$ = gate-source voltage, I${}_{DS}$ = drain-source current, and pHEMT = pseudomorphic high electron mobility transistor.
